# Supplementary material for: Trappc9 Deficiency Impairs the Plasticity of Stem Cells
Source: Int J Mol Sci. 2022 Apr 28;23(9):4900. doi: 10.3390/ijms23094900 (PMC9101649; doi:10.3390/ijms23094900)
Supplement: Supplementary file 1 [file ijms-23-04900-s001.zip › ijms-1641546-supplementary.pdf]

Supplementary figures and figure legends

Figure S1

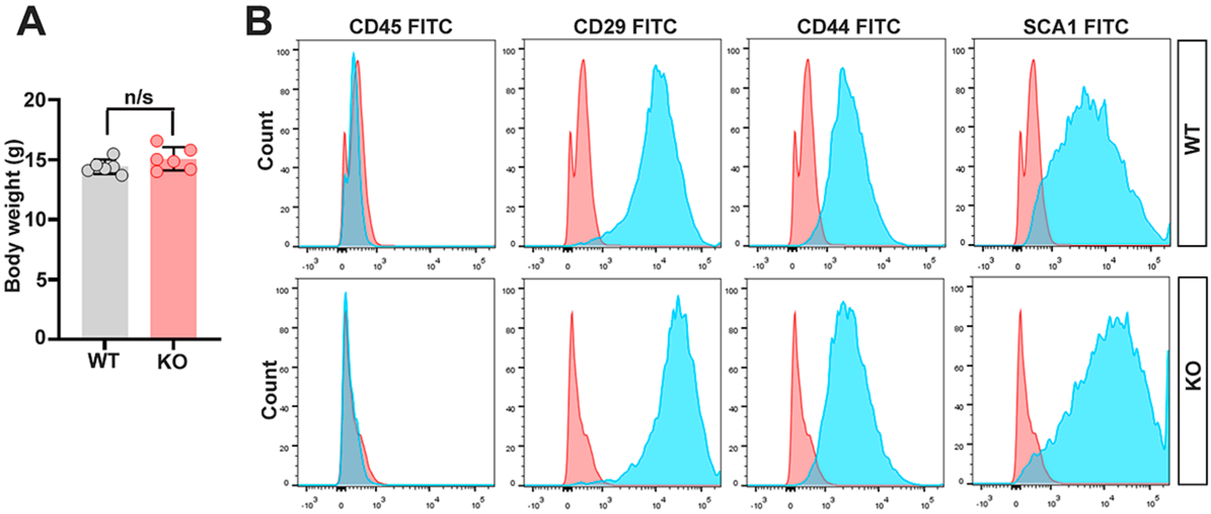

Figure S1 Isolation and characterization of adipose-derived stem cells. A) The body weight of trappc9-deficient (**KO**) mice used for isolating stem cells from abdominal adipose tissues was similar to that of WT mice. **B**) Flow cytometry analysis showed that both WT and KO ASCs at passage-3 expectedly expressed protein markers specific for mesenchymal stem cells and did not express pan-hematopoietic protein marker CD45.

Figure S2

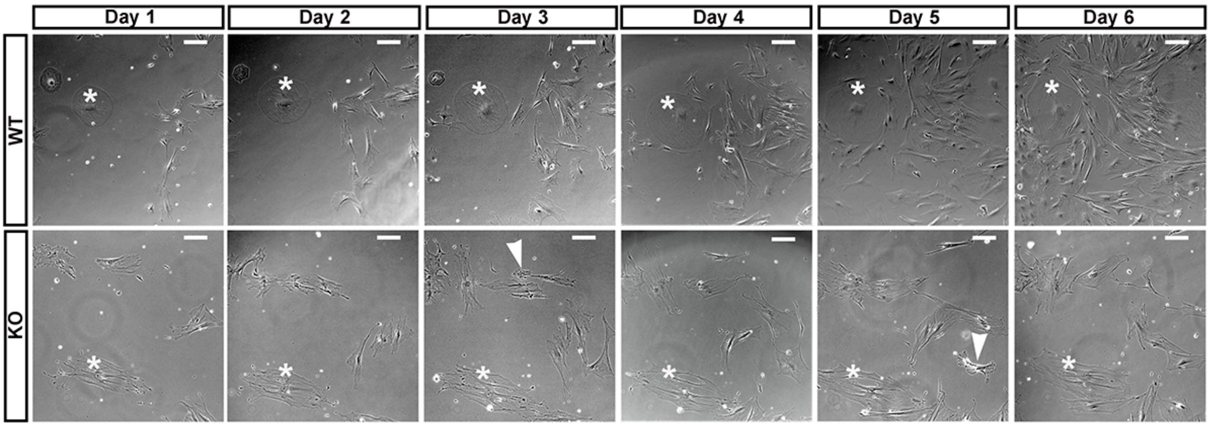

Figure S2 Loss of cells in trappc9-deficient ASC cultures at very early stages. WT and trappc9-deficient (**KO**) ASCs at passage-4 were cultured in T25 flasks at a density of $1x10^5$ and examined every 24 hours under an Olympus IX73 inverted microscope. Images were captured from the same locus, which was demarcated outside the flasks. Stars indicated the same cell captured over 6 days. Arrowheads pointed to cells which disappeared in the following day. Scale bars: 100μm
